# Adsorption Sites in the High-Coverage Limit of CO on Cu(111)

**DOI:** 10.1021/acs.jpcc.4c07044

**Published:** 2025-02-06

**Authors:** Diyu Zhang, Vladyslav Virchenko, Charlotte Jansen, Irene M. N. Groot, Ludo B. F. Juurlink

**Affiliations:** † Leiden Institute of Chemistry, Leiden University, PO Box 9501, 2300 RA Leiden, The Netherlands; ‡ School of Science, Key Laboratory of High Performance Scientific Computation, Xihua University, Chengdu 610039, China

## Abstract

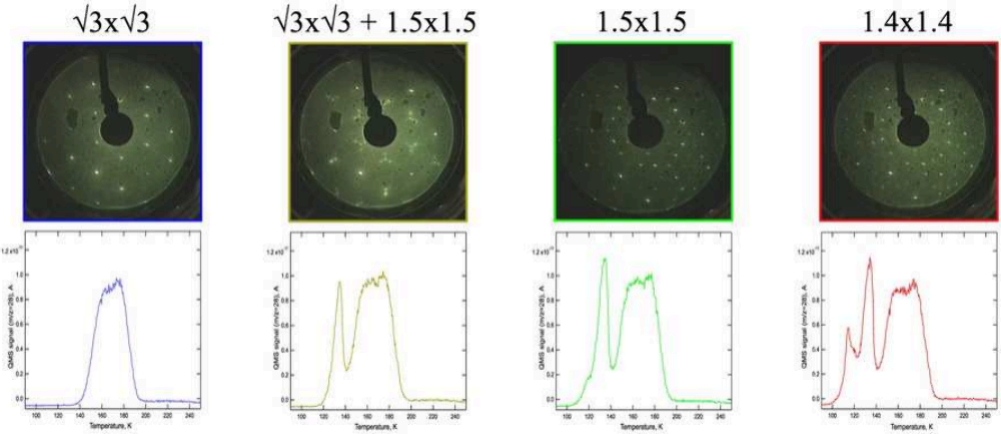

The development of a comprehensive theory describing gas-surface interactions requires accurate experimental data for benchmarking. The adsorption of CO to Cu(111) is such a benchmark system. While state-of-the-art calculations still erroneously predict the favored adsorption site at low coverage to be the 3-fold hollow site, experimental studies have not yet definitively identified adsorption sites for all overlayer structures. Using a new combination of well-established techniques, we have reinvestigated CO adsorption, its ordering, and desorption on Cu(111). Our results support earlier suggestions for various overlayer structures for different coverages. For the intermediate-coverage 1.5×1.5 structure, we show that only on-top sites are occupied. Bridge site adsorption occurs only beyond a coverage of 0.42 monolayer (ML) in the 1.4×1.4 structure. Occupancy of this site is associated with a shift of atop CO molecules to off-centered atop with a characteristic IR absorbance frequency. These findings indicate a complex balance of coverage-dependent adsorbate interactions and binding energies that results in nonintuitive ordering and requires improvements in theory to understand.

## Introduction

Even though the adsorption of molecules on metal surfaces has been studied for a long time (for a comprehensive overview, see the book of Díez Muiño and Busnengo^1^), new findings still occur and a universal theory for the understanding of gas-surface dynamics has not been developed yet. Such a theory would be of great relevance to fields of, e.g., surface science, heterogeneous catalysis, two-dimensional materials growth, and tribology.

While major steps have been taken in developing such a theory, state-of-the-art approaches still incorrectly predict the preferred adsorption site for the benchmark system CO/Cu(111).^2−6^ All theoretical studies predict that in the lower coverage regime CO binds to 3-fold hollow sites. Experimental studies have explored CO adsorption and the various overlayers formed at different coverages on flat Cu(111) single crystals since (at least) 1972 and show that CO adsorption occurs primarily atop.^7−12^ These studies mostly involved surface potential measurements, reflection absorption infrared spectroscopy (RAIRS), scanning tunneling microscopy, and low-energy electron diffraction (LEED). They identified three overlayer structures, i.e., (√3×√3)*R*30° (with a maximum coverage of 0.33 monolayer (ML)), (1.5×1.5)*R*18° (0.42 ML), and (1.4×1.4) (0.52 ML).^7,8,13,14^ These numbers reflect the average CO–CO nearest distance along the edges of the overlayer’s unit cell.

For the (√3×√3) structure, only atop adsorption occurs as inferred from a single IR absorbance initially occurring at 2078 cm^–1^.^15−20^ A nonlinear coverage-dependent redshift of the central frequency and increase in line width have been reported in various studies.^15,16,20^ The redshift has been ascribed to a balance between dipole interactions and the CO-Cu binding energy with opposite effects on the internal CO stretch frequency. While for Pt(111) this balance leads to a blueshift, the opposite occurs on Cu(111). The redshift with increasing coverage is also opposite to the predicted shift from increasing coverages of DFT-based calculations for CO/Cu(111).^21^ This, again, points to a lack of accuracy in the current theoretical description of the adsorption of gases to surfaces.

The interpretation of the more complex (1.5×1.5) and (1.4×1.4) structures has been debated as multiple real-space structures may underly the observed diffraction patterns.^7,8,13,14,16,22,23^ For the (1.4×1.4) structure, a recent low-temperature scanning tunneling microscopy (STM) study showed a real-space pattern with atop and bridge occupancy only.^24^ This pattern is nearly the same as the nonuniformly compressed structure originally proposed for the (1.4×1.4) LEED pattern by Pritchard and Hollins.^8^ The suggestion of nonuniform compression of the overlayer stemmed in part from a weak double IR absorption appearing at 1835 and 1814 cm^–1^ with increasing coverage.^19^ This frequency range is typical of bridge-site adsorption. Combined with the weak redshift for the atop absorbance, these spectral features suggest that CO molecules tend to bind to atop and (at high coverage) to bridge site positions. Uniform compression would require gradual shifts from these positions.

The real space structure underlying the (1.5×1.5) LEED pattern has not been resolved by STM. The interpretation as a uniformly compressed and rotated (√3×√3)-type structure does not seem plausible as, somewhere in the relevant coverage range, the redshifting atop IR absorption is joined by the final double bridge IR absorptions. The exact coverage at which the bridge adsorption appears in IR spectra has not been defined. A study using molecular beam techniques for highly controlled CO adsorption, related adsorption characteristics to desorption from temperature-programmed desorption (TPD) spectra.^25^ This study suggests that in the (1.5×1.5) structure, bridge sites are occupied. In this study, we have used a combination of molecular beams, temperature-programmed desorption (TPD), LEED, and RAIRS to resolve whether this is the case. Our results conclusively allow us to relate the IR frequency of CO to the exact locations of binding. This provides a benchmark for theoretical underpinning of gas-surface interactions which is - as of yet - still incapable of correctly predicting the adsorption site(s) and the frequency effects of coverage to the internal CO bond. In particular, the two frequencies for ”centered on-top” and ”off-centered on-top” have never been reported before and are essential to the understanding how binding and molecular orbital overlap between CO and the Cu substrate affect the internal CO electronic structure and, hence, IR frequency.

## Experimental Methods

The data were obtained in an ultrahigh vacuum (UHV) system with a base pressure of ≈2.0 × 10^–10^ mbar. The system is pumped down by a standard combination of turbomolecular pumps backed by rotary vane oil pumps with an oil filter in between to avoid contamination of the chambers. The main chamber is equipped with Auger electron spectroscopy (DESA2000, STAIB instruments), low-energy electron diffraction (erLEED 1000-D, Vacuum Science Instruments), and a quadrupole mass spectrometer (QME200, Pfeiffer). Residual gas analysis (RGA) is performed daily to ensure a stable residual gas composition. The sample is welded to a copper block attached to the XYZ-manipulator with a coldfinger and a differentially pumped rotary platform. A filament is placed approximately 1 mm away from the back side of the sample. Thus, the sample temperature is controlled within a range of 80–800 K, measured with a K-type thermocouple welded directly to the base of the sample itself. A differentially pumped QMS used for temperature-programmed desorption (TPD) measurements is placed inside the main chamber facing the sample with the orifice slightly smaller than the sample itself to ensure better signal intensity. Standard gases (Ar, CO, H_2_, O_2_) are introduced through precision leak-valves followed by an RGA measurement. The sample is grounded and connected to a high-precision automatic picoammeter (Keithley 485).

Sample preparation was performed by cycles of 10 min of Ar^+^-sputtering (0.8 kV energy, 8 mA emission) at elevated temperature (400 K), followed by subsequent 10 min of annealing at 800 K. Sample cleanliness was confirmed by means of LEED and TPD.

## Results and Discussion

We adsorb CO onto the Cu(111) surface (T_*surf*_ = 100 K) using the highly stable flux from a He-seeded molecular beam. Since CO has a very high sticking probability on Cu(111), using a pure CO beam would lead to instantaneous saturation of the surface with CO. To be able to record an accurate King and Wells curve,^26^ we have seeded CO in He. In addition, when seeding CO in He, the lower mass of He compared to CO results in a higher velocity, and, hence, a higher kinetic energy of the CO molecules being adsorbed onto the Cu(111) surface.^27^ We use a molecular beam of 1% CO in He. The kinetic energy of the CO molecules can be estimated by the following equation:^27,28^
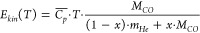
1where *C̅*_*p*_ is the molar average heat capacity for the gas mixtures, *M*_*CO*_ and *m*_*He*_ are CO and He molecular weights, and T is the nozzle temperature.

**Figure 1:**
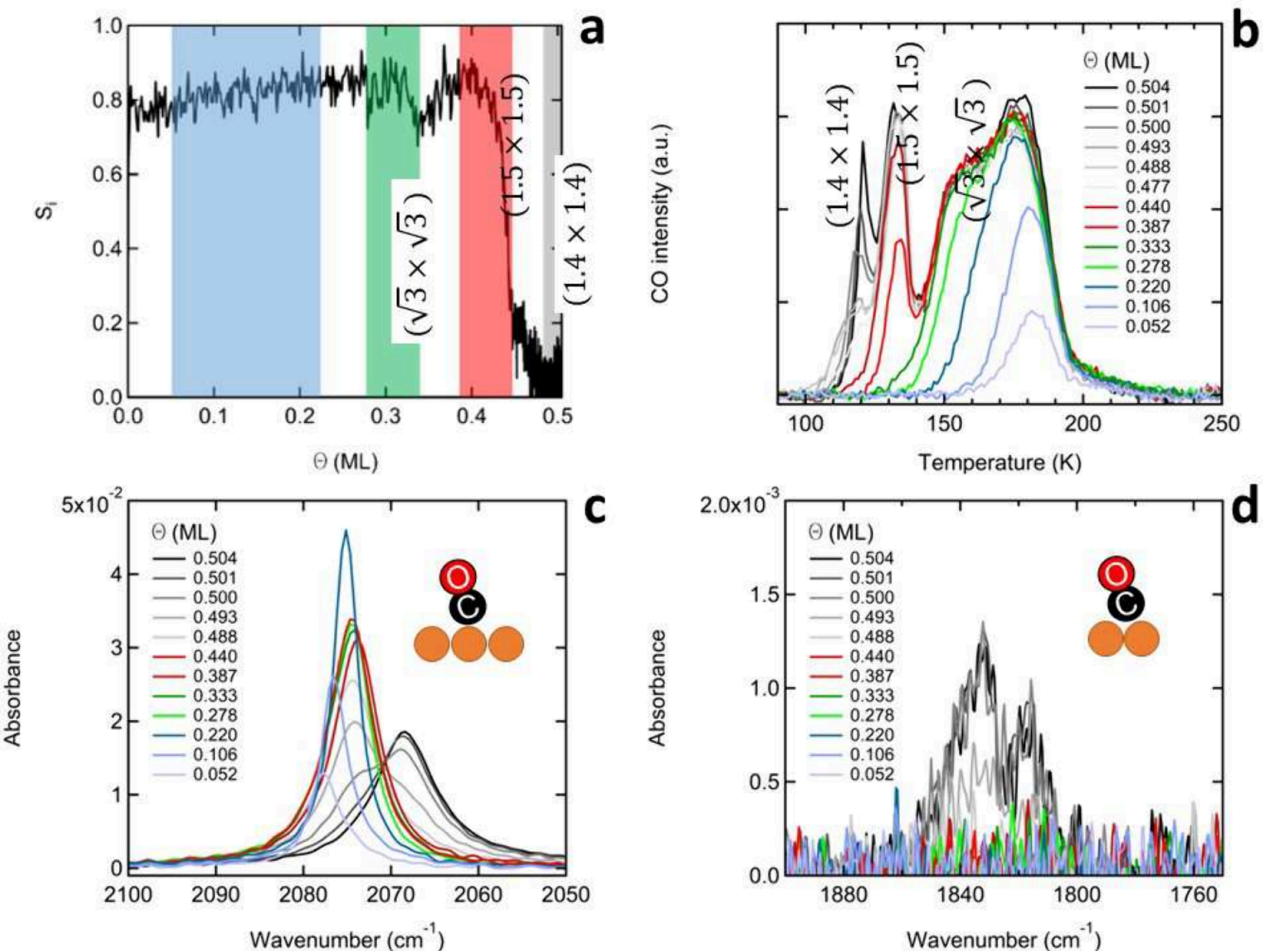
a) Sticking probability of CO on Cu(111). Indicated in blue, green, red, and gray are the different structures of the CO overlayer for different coverages. b) TPD spectra after CO deposition for different coverages. CO is adsorbed onto the surface from a molecular beam. c) RAIR spectra for the frequency range of atop adsorption of CO on Cu(111). d) RAIR spectra for the frequency range of bridge adsorption of CO on Cu(111).

We track the sticking probability as a function of time. A series of these experiments with varying exposure times are followed by TPD experiments. The Supporting Information (Section S1) provides details on how the integration of King and Wells traces allows us to convert exposure time to the attained coverage. Figure 1a shows the sticking probability for a beam of CO molecules with a kinetic energy of 429 meV (obtained from eq 1) as a function of coverage until saturation is achieved.

Figure 1b shows a set of TPD spectra. The colors of the TPD traces reflect the known overlayer structures and the colored boxes in Figure 1a. Up to 0.25, 0.33, 0.42, and 0.52 ML, we use shades of blue, green, red, and gray/black, respectively. Corresponding LEED patterns are shown in the Supporting Information, Figure S1.

The initial sticking probability is around 0.8, which up to around 0.3 ML of CO slowly increases to around 0.9. It then shows a characteristic dip.^25,29^ Our data confirm that the minimum occurs at the completion of the (√3×√3) structure. Subsequent formation of the (1.5×1.5) structure is, at first, facile. After the recovery of the high sticking probability, it plummets, though, near the completion of this overlayer structure. Formation of the (1.4×1.4) structure starts with another discontinuity in the sticking probability.^25^ Completion of this structure, however, is difficult throughout, as the sticking probability remains very low.

The TPD spectra in Figure 1b confirm that the presence of various overlayer types is distinguishable by clear features in the spectra. Up to 0.25 ML, CO desorbs in a single feature peaking near 170–180 K (blue spectra). In between 0.25 and 0.33 ML, CO desorbs in a shoulder at the low-temperature side of this peak (green spectra). Beyond 0.33 ML, the (√3×√3) structure disintegrates. The desorption of the (1.5×1.5) structure is observed as a characteristic second and well-defined peak at ≈132 K (red spectra). Decomposition of the (1.4×1.4) structure occurs by desorption around 120 K (gray spectra).

Having related adsorption and desorption features to coverage, we turn to the dependence of IR spectral features on coverage. Figure 1c and 1d shows the development of the absorbances in the respective frequency ranges for atop and bridge positions after deposition of CO onto Cu(111) by our molecular beam. We use the same colors for coverage as before, i.e., 0.25 (blue), 0.33 (green), 0.42 (red), and 0.52 ML (black). Others are shown in shades of gray. In the atop region, the well-defined absorbance starting at 2078 cm^–1^ drops to 2075 cm ^–1^ and increases in intensity up to 0.25 ML. The initial formation of the √3×√3 structure is signaled by a sudden drop in intensity and minor broadening, while the redshift in peak frequency stalls at 2074 cm^–1^.^19^ The peak then hardly changes up to a coverage of 0.42 ML.

Previously, further development of the absorbance was described as continued redshifting and/or distortion from the symmetric shape without highly accurate connection to coverage or overlayer structures.^17,19^Figure 1d couples the onset of the final changes in the atop region to the appearance of the absorbances signaling bridge-site occupation. Only beyond the completion of the (1.5×1.5) structure, hence the start of the (1.4×1.4) structure, do we observe the two absorbances centered near 1835 and 1814 cm^–1^.^19^ The appearance of these peaks is accompanied by the distortion of the symmetric peak in the atop region (Figure 1c).

**Figure 2:**
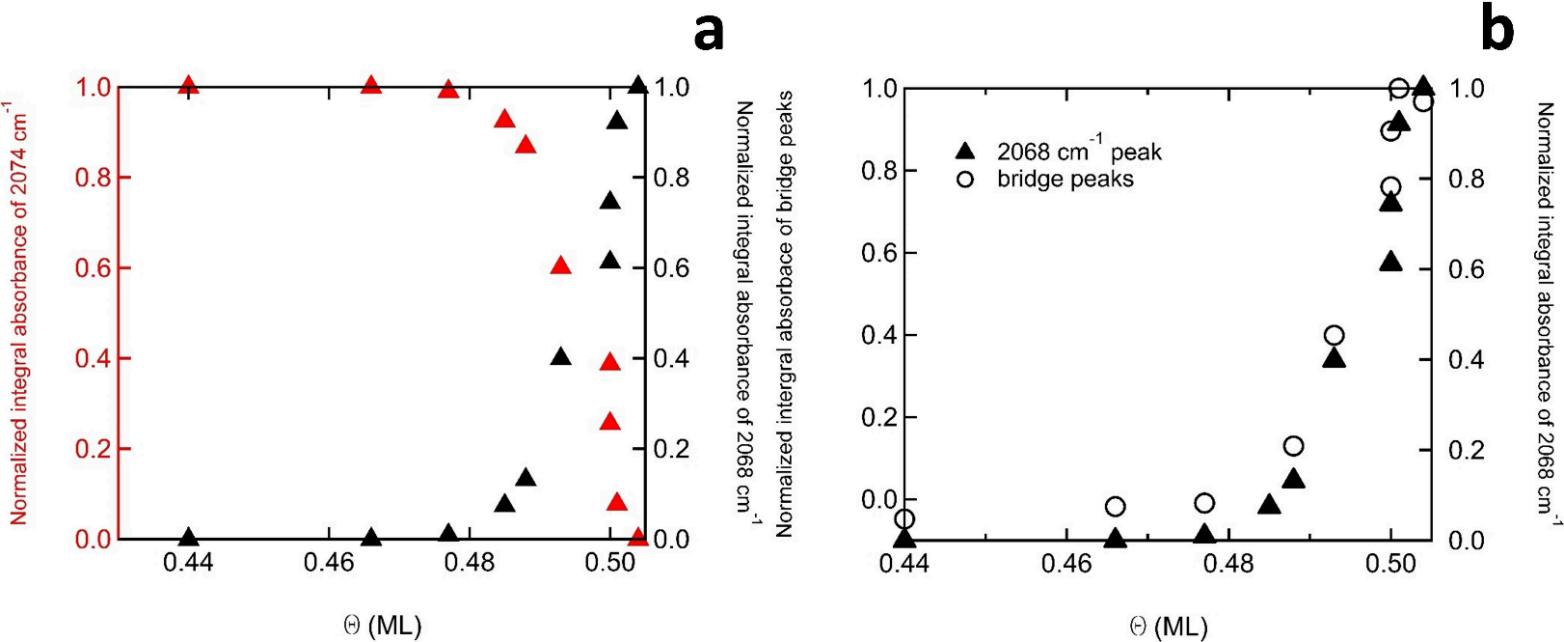
a) Normalized integral absorbance of the 2074 cm^–1^ feature of atop-bound CO versus coverage (red, left *y*-axis) and of the 2068 cm^–1^ feature (black, right *y*-axis). b) Normalized integral absorbance of the feature of bridge-bound CO versus coverage (left *y*-axis) and of the 2068 cm^–1^ feature (right *y*-axis).

We find that the spectra in the atop region beyond 0.42 ML are better explained as a sum of two independent absorbances centered at 2075 and 2068 cm^–1^, respectively. We have applied a fitting procedure representing a linear combination of two absorbances centered at these frequencies and show the results in Figure 2a. The maximum integrated absorbances are normalized using the two peaks observed at 0.44 and 0.504 ML in Figure 1c. The resulting data show that the loss of the peak at 2074 ^–1^ (red triangles) can be quantitatively related to the appearance of an independent absorbance at 2068 cm^–1^ over the coverage regime where the (1.4×1.4) structure develops from the (1.5×1.5) structure. The new absorbance therefore represents atop CO that is different from atop CO occurring in the (1.5×1.5) structure. This could be by the exact location (e.g., off-centered atop) or by the structure of the CO molecules forming its surroundings. Figure 2b plots the same data for the appearance of the 2068 cm^–1^ absorbance as a function of coverage (right *y*-axis) with the integrated absorbance of both features in the frequency regime for bridge-bound CO (left *y*-axis). These data support that the new atop feature is directly connected to the appearance of the bridge-bound CO molecules. Independent integration of the two absorbances in the latter regime, i.e. those at 1835 and 1814 cm^–1^, suggests these two features develop simultaneously.

Our results help clarify aspects of both higher-coverage structures ((1.5×1.5) and (1.4×1.4)). First, for the final structure at 0.52 ML coverage, nonuniform compression and rotation of the (√3×√3) structure suggested from LEED studies by Hollins and Pritchard^13^ was shown to occur by the real-space study of the (1.4×1.4) structure.^24^ The STM images showed stripes of sole bridge sites that appear lower than stripes containing a mix of top and bridge sites. The proposed unit cell for this structure contains 49 Cu atoms (7×7) and 25 CO molecules. It seems reasonable that the two different types of bridge sites in this structure (each occurring with 6 out of 25 CO molecules, hence a total of 12 bridge-bound CO molecules) are responsible for the two absorbances at 1835 and 1814 cm^–1^. Off-centered atop CO occurs in groups of 4 CO molecules each and also a total of 12 out of 25 CO molecules per unit cell. These would most likely be responsible for the 2068 cm^–1^ IR absorbance. The final single centered-atop CO molecule in this structure is, apparently, not stealing IR intensity from the lower frequency of the 12 off-centered atop CO molecules to an extent that it remains clearly visible as a separate feature in the spectra at 2074 cm^–1^.

For the site occupancy of the (1.5×1.5) structure, our data can be argued to support Hollins and Pritchard’s proposed unit cell.^13^ As there is no indication of bridge site occupancy, CO molecules are assumed to be bound atop only. On 16 Cu atoms (4×4), 7 atop CO molecules (all centered) are divided over 2 clusters of 3 CO molecules interspersed by single CO molecules (1 per unit cell). The invariant IR absorbance in the regime of 0.33–0.44 ML must then be interpreted to indicate that either reducing the distance between CO molecules to closer than √3 does not affect their absorption frequency or that CO clusters with a lowered absorbance are invisible due to intensity-stealing by the lone CO molecules. The latter seems highly unlikely as in the highest coverage structure discussed above we find no evidence of the lone CO’s in a ratio of 1:12 in comparison to the clustered, but off-centered, atop CO’s appearing at 2068 cm^–1^. Hence, it appears that the CO internal stretch frequency does not vary with CO–CO distance below √3, but is affected when forced into an off-centered atop position. At the same time, the appearance of a new desorption feature in the TPD spectra argues for lowered binding energy. This point reiterates that the relation between the CO binding energy and internal C = O vibrational frequency are not properly understood. This may also be taken to indicate that development of a more accurate description of the interaction of CO with metallic surfaces is still highly needed.

## Summary and Conclusion

Using a combination of molecular beams, RAIRS, TPD, and LEED, we have consolidated the experimental picture of CO adsorption on Cu(111). We observe three different CO adsorption structures depending on coverage: the (√3×√3) structure up to 0.33 ML, with a desorption temperature of 170–180 K and the RAIRS signature of atop bonding at 2074 cm^–1^; the (1.5×1.5) structure with a CO coverage of 0.44 ML, desorbing at 132 K and with atop bonding as indicated by the RAIRS feature at 2074 cm^–1^; and the (1.4×1.4) structure with a coverage of 0.52 ML and desorption at 120 K. Here, CO is both bound off-centered atop (2068 cm^–1^) and in bridge positions (1835 and 1814 cm^–1^). Whereas initiation and growth of the (1.5×1.5) phase within the (√3×√3) phase is rather facile, the same does not hold for the (1.4×1.4) phase within the (1.5×1.5) phase. The low sticking probability beyond the 0.44 ML coverage points toward a significant energy barrier for formation of the highest coverage phase, which is then also reflected in a very low temperature for CO desorption in the final coverage regime. Finally, we have shown that a reinterpretation of the IR absorbance by the atop site is crucial for a complete picture of CO adsorption on Cu(111), ranging from low to high coverage.

## Supporting Information Available

Integration of TPD spectra to obtain CO coverages on Cu(111) and LEED patterns for the different CO adsorption structures on Cu(111) (PDF)
