# Supplementary material for: Adsorption Sites in the High-Coverage Limit of CO on Cu(111)
Source: J Phys Chem C Nanomater Interfaces. 2025 Feb 6;129(7):3493–7. doi: 10.1021/acs.jpcc.4c07044 (PMC11848902; doi:10.1021/acs.jpcc.4c07044)
Supplement: Supplementary file 1 — jp4c07044_si_001.pdf [file jp4c07044_si_001.pdf]

SUPPORTING INFORMATION for Adsorption Sites in the High-Coverage Limit of CO on Cu(111)

Diyu Zhang,[†,‡] Vladyslav Virchenko,[†] Charlotte Jansen,[†] Irene M.N. Groot,[†] and Ludo B.F. Juurlink[*,†]

†*Leiden Institute of Chemistry, Leiden University, PO Box 9501, 2300 RA Leiden, the Netherlands*

‡*School of Science, Key Laboratory of High Performance Scientific Computation, Xihua University, Chengdu 610039, China*

E-mail: l.juurlink@chem.leidenuniv.nl

Phone: +31 (0)71 5274221

S1: TPD integration and coverage

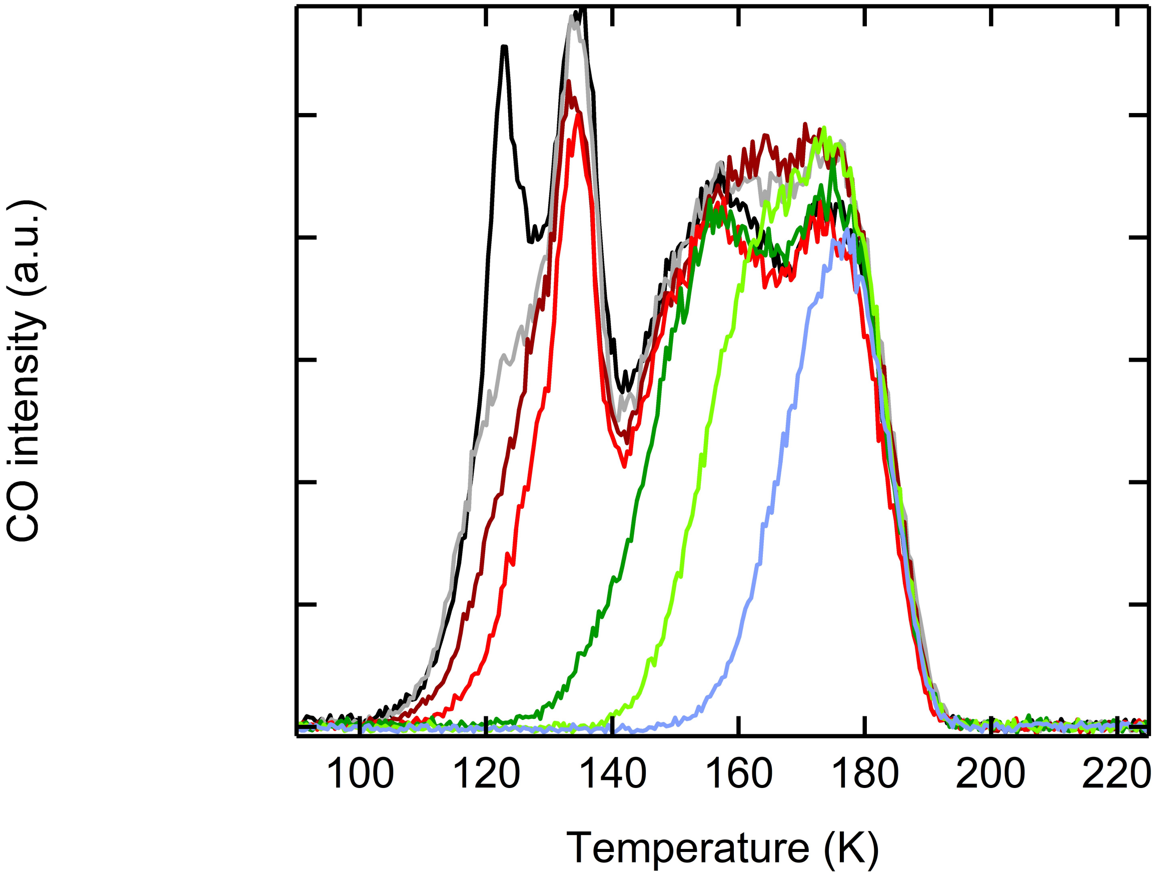

Figure 1: Raw data for several TPD traces at different CO coverages. From black/gray to red to green to blue, the coverage of CO on the surface decreases.

In Figure S1, we show the raw data for several TPD traces, taken at different coverages of CO on Cu(111). Going from black/gray to red to green to blue, the coverage of CO on the surface decreases, from 0.504 ML to ≈0.14 ML. In general, it is possible to obtain the absolute coverage of CO on the surface from intergrating TPD peaks. However, the sticking probability of CO on Cu(111) in UHV is too high to quickly pump CO away. Therefore, the background of CO will be quickly built up, which affects the accuracy of our data. Therefore, we make use of our King and Wells measurements instead. In the King and Wells experiments, there are two flags to control the CO molecular beam. The first one is before the measurement chamber, the second one is in front of the Cu(111) sample. Here, we

keep flag 1 on, which allows the CO molecular beam to continuously enter the measurement chamber. Then, we keep switching on and off flag 2. We measure the King and Wells curve and check the background level at the same time, see Fig. S2, left panel. We fit the data recorded while flag 2 is off (yellow line). This intensity contains the intensity of CO coming directly from the molecular beam, the intensity of the CO background, and the pumping speed difference when flag 2 is on or off. First, we rule out the pumping speed factor and get the green line in Fig. S2, left panel. We then remove the data measured when flag 2 is off, and this results in the sticking probability of CO on Cu(111), see the black curve in Fig. S2, right panel. We then calculate the integral of this curve, which is the measured absolute coverage of CO on the surface, and has a value of 0.504 ML, see Figure 1 of the main manuscript.

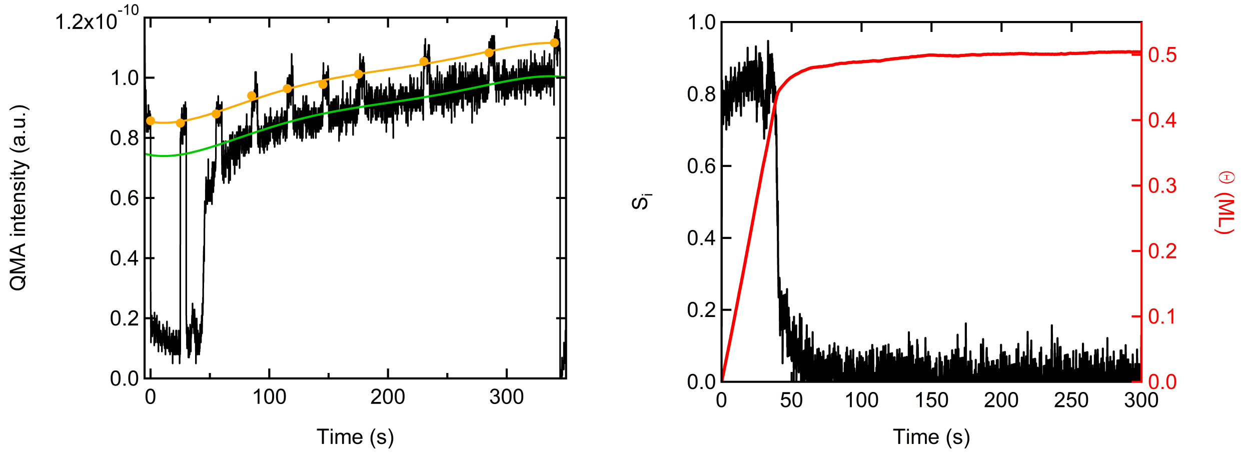

Figure 2: Left: King and Wells measurement with switcing on and off flag 2. Right: King and Wells trace of CO adsorption on Cu(111) and the obtained maximum coverage.

S2: LEED study of CO on Cu(111)

Using the setup described in the main text, we have studied adsorption structures by LEED. Figure S3 shows an overview of our results for various coverages of CO on the Cu(111) surface. In between every experiment the surface is cleaned using our standard sputter-anneal cycles. CO was dosed via a leak valve onto the Cu(111) surface held at 80 K. The

copper surface was exposed to a significant amount of CO to ensure full saturation. Then, the sample was heated to a particular temperature (110 K, 125 K, 145 K, 165 K, and 210 K) in order to remove the (1.4×1.4),(1.5×1.5),$(\sqrt{3} \times \sqrt{3})$, and (2×2) structures, respectively. Figure S3 shows the resulting LEED patterns. Surprisingly, the collected LEED patterns do not show a clear co-existence of the (1.5×1.5) and $(\sqrt{3} \times \sqrt{3})$ structures, but rather only the $(\sqrt{3} \times \sqrt{3})$ structure. The (2×2) structure was not observed.

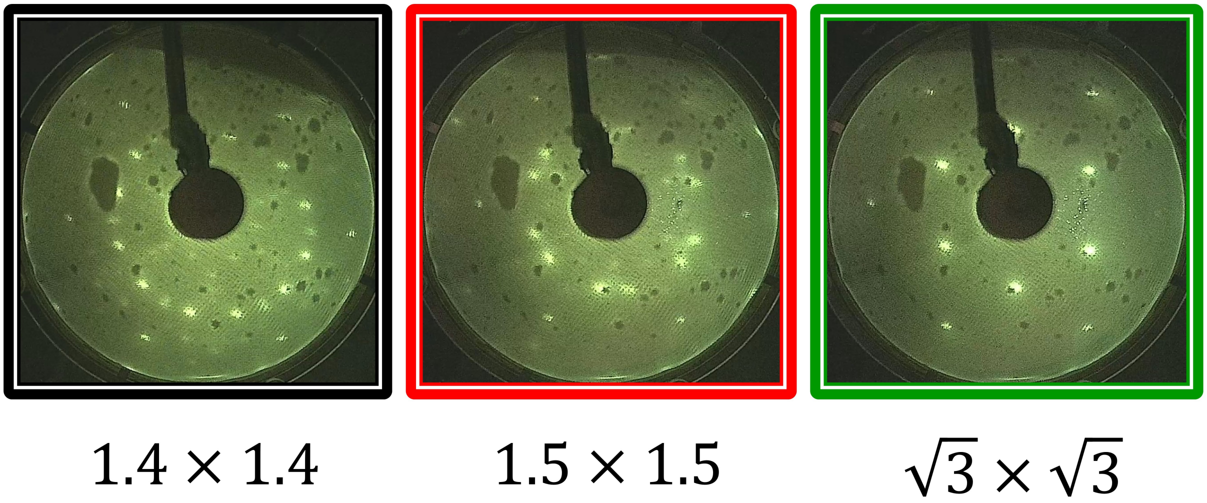

Figure 3: LEED patterns of (1.4×1.4), (1.5×1.5), and $(\sqrt{3} \times \sqrt{3})$ structures (left to right). The color coding corresponds to Figure 1 in the main text.
